# Clinical and Pathophysiological Considerations Related to the Impact of Bulevirtide, a New Entry Inhibitor, in HBV-HDV Infection

**DOI:** 10.3390/v18040477

**Published:** 2026-04-19

**Authors:** Raisa Eloise Barbu, Mariana Daniela Ignat, Roxana Elena Bogdan Goroftei, Alexia Anastasia Ștefania Baltă, Valerii Lutenco, Valentin Bulza, Valerian Ionuț Stoian, Simona Claudia Cambrea, Elena Dumea, Liliana Baroiu

**Affiliations:** 1 Doctoral School of Biomedical Sciences, ‘Dunărea de Jos’ University of Galați, 800008 Galați, Romaniavalerii.lutenco@ugal.ro (V.L.);; 2 Faculty of Medicine and Pharmacy, Research Centre in the Medical-Pharmaceutical Field, ‘Dunărea de Jos’ University of Galați, 800008 Galați, Romania; 3 Clinical Medical Department, Faculty of Medicine and Pharmacy, ‘Dunărea de Jos’ University of Galați, 800008 Galați, Romania; 4 ‘Sf. Ioan’ Clinical Hospital for Children, 800487 Galați, Romania; 5 ‘Sf. Cuv. Parascheva’ Clinical Hospital of Infectious Diseases, 800179 Galați, Romania; 6 Depatment of Morphofunctional Science, Faculty of Medicine and Pharmacy, ‘Dunărea de Jos’ University of Galați, 800008 Galați, Romania; 7 ‘Sf. Apostol Andrei’ Clinical Emergency County Hospital, 800578 Galați, Romania; 8 Medical Department, Faculty of Medicine and Pharmacy, ‘Dunărea de Jos’ University of Galați, 800008 Galați, Romania; 9 Clinical Surgical Department, Faculty of Medicine and Pharmacy, ‘Dunărea de Jos’ University of Galați, 800008 Galați, Romania; 10 Galați Railways General Hospital, 800223 Galați, Romania; 11 National Institute for Public Health, 050463 Bucharest, Romania; 12 Faculty of Medicine, ‘Ovidius’ University, 900470 Constanta, Romania; 13 Clinical Hospital of Infectious Diseases, 900178 Constanta, Romania

**Keywords:** HBV/HDV coinfection, bulevirtide, viral entry inhibition

## Abstract

This review critically examines the inhibition of viral entry as an emerging disease-modifying strategy in chronic hepatitis B (HBV) and delta (HDV) virus infection, with particular emphasis on bulevirtide, the first-in-class of the sodium taurocholate cotransporting polypeptide entry inhibitor. This paper summarizes the analysis of 7 clinical trials that either underpinned the registration of bulevirtide or are important European real-life trials. We synthesize virological, pathophysiological and clinical evidence, highlighting the impact of this novel bulevirtide-based therapy on virological control, liver inflammation, fibrosis dynamics and long-term prognosis, as well as the limitations of this therapy. The observation of these trials is a greater than 2 log decrease from baseline in hepatitis D virus ribonucleic acid (HDV RNA) in 54–92% of patients and normalization of alanine transaminase (ALT) in 48.8–74% of patients after 23–144 weeks of treatment, and a significant decrease in liver fibrosis, as quantified by Fibroscan, at 12 months of treatment. The conclusion of the study is that this therapy represents an important leap in the etiological approach to chronic HDV infection and in improving the prognosis of these patients, but future clinical studies are needed to define the criteria for discontinuation of therapy, the long-term impact, as well as studies targeting new therapies that can intervene in other stages of the HDV and HBV life cycle not only to achieve HDV RNA negativity but also HBsAg clearance.

## 1. Introduction

Chronic hepatitis B virus infection continues to represent a substantial global health concern, affecting 254 millions people (according to the World Health Organization, in 2022) and remaining a major contributor to liver-related morbidity and mortality worldwide [1,2,3]. Although universal vaccination programs have significantly reduced incidence in many regions, and potent nucleos(t)ide analogues provide effective suppression of viral replication, HBV infection persists as a complex clinical problem. This is particularly true for individuals with advanced liver disease or concomitant viral infections, in whom disease management is more challenging and outcomes are often less favorable [4,5,6].

Hepatitis D virus is an incomplete RNA virus that cannot propagate independently, as it requires the presence of HBV to complete its life cycle. In particular, HDV depends on HBsAg for the assembly and release of new virions, as well as for entry into hepatocytes [7,8,9]. This biological interdependence creates a close virological and clinical relationship between HBV and HDV, whereby liver injury results not only from viral replication itself but also from an intensified immune response directed against infected hepatocytes [10,11,12].

Worldwide, it is estimated that there are between 48 and 60 million patients with chronic hepatitis with HDV with a prevalence of 0.80% in the general population (95% confidence interval [CI], 0.63–1.00). The prevalence of patients with chronic hepatitis with HDV among patients chronically infected with HBV is 13.02% (95% CI, 11.96–14.11) [13]. Data from epidemiological and clinical investigations consistently indicate that individuals with HBV/HDV infection face a greater risk of cirrhosis (with odds ratio (OR) of 3.84 (95% CI, 1.79–8.24)), hepatic decompensation, and hepatocellular carcinoma (HCC) than those with HBV infection alone [14,15,16,17,18,19]. Clinical studies have observed a progression rate of chronic HBV/HDV infection to cirrhosis within 5 years and to HCC within 10 years, on average, in the absence of etiological treatment [15].

Historically, therapeutic options for HBV/HDV infection have been extremely limited. While nucleos(t)ide analogues effectively suppress HBV DNA replication and mitigate HBV-associated complications; r, they however exert limited direct influence on HDV replication or on the clinical course of HDV-related disease [5,18,20]. European Association for the Study of the Liver (EASL) 2017, guidelines recommend Pegylated interferon-alpha (Peg IFN alpha) ≥48 weeks for chronic hepatitis HBV/HDV and compensated liver cirrhosis and contraindicate this treatment in decompensated liver cirrhosis [21]. American Association for the Study of Liver Diseases (AASLD) 2018, guidelines recommend Peg IFN alpha for 1 year in patients with HDV RNA and elevated ALT [22]. World Health Organization (WHO) 2021, guidelines recommend Peg IFN alpha ≥48 weeks, regardless of patient response, with contraindications for decompensated cirrhosis, psychiatric conditions or autoimmune diseases [23]. Thus, Peg IFN alpha has historically constituted the sole approved therapeutic option for HDV infection; nevertheless, its limited antiviral efficacy, suboptimal tolerability profile, numerous contraindications, and high rates of post-therapy relapse have substantially constrained its clinical applicability [24,25,26].

The first guideline that introduced as a therapeutic alternative besides Peg IFN alfa, bulevirtide, in 2021, was European AIDS Clinical Society (EACS) for patients with advanced liver fibrosis [27]. The bulevirtide, a first-in-class viral entry inhibitor that targets the sodium taurocholate cotransporting polypeptide (NTCP), represents a significant therapeutic advance in the management of HBV/HDV infection [28,29]. By blocking viral entry at the hepatocyte level, bulevirtide disrupts a critical shared step in the life cycle of both HBV and HDV, offering a mechanism of action distinct from conventional replication-directed antiviral strategies [30,31].

In July 2023, bulevirtide received unconditional approval from the European Medicines Agency (EMA) [32] and the EASL 2023 guideline recommends bulevirtide as monotherapy or in combination with Peg IFN alfa in patients who are not intolerant or who have no contraindications to Peg IFN alfa [33].

This article explores the clinical and pathophysiological impact of targeting viral entry in HBV/HDV infection, with a particular focus on bulevirtide-based therapy. Drawing exclusively on data synthesized in the present article, we examine the virological rationale, clinical impact, safety profile, and prognostic implications of this approach. Rather than focusing solely on HDV, the discussion addresses HBV/HDV infection as a unified pathological entity, highlighting the broader implications of entry inhibition strategies for the management of complex chronic viral hepatitis.

## 2. Materials and Methods

We conducted searches in the databases: PubMed, Google Scholar and Research Gate and examined meta-analyses and clinical studies from 2019 to 2026, using keywords such as HDV, treatment, antiviral therapies, clinical studies, bulevirtide. The inclusion criteria were: multicenter studies that include more than 50 patients with chronic HBV and HDV infection for over 23 weeks, studies that used antivirals including bulevirtide and studies with full-text availability. The exclusion criteria were: studies that include patients with hepatitis C virus coinfection, alcoholic liver disease and fatty liver, patients under 18 years old. Out of the total 3084 articles, after excluding duplicates and articles that did not meet the inclusion criteria, were left 7 clinical studies. For therapeutic indications, the guidelines referring to this treatment were analyzed.

## 3. Mechanistic Rationale for Viral Entry Inhibition in HBV/HDV Infection

Hepatitis D virus is a small, defective, circular, single-stranded RNA virus characterized by an absolute dependence on hepatitis B virus for the completion of its life cycle [34,35]. The structure of HDV includes a negative-strand RNA genome of 1672–1697 nucleotides, depending on the genotype. This genome encodes two protein isoforms, termed small (S-HDAg) and large (L-HDAg) Delta antigen, which mediate genome replication and virion assembly by acting as nucleoproteins [36,37,38]. HDV is dependent on HBsAg for packaging its ribonucleoprotein complex. HBV encodes three envelope proteins, namely HBsAg small (S-HBs), medium (M-HBs) and large (L-HBs), which are encoded by a single open reading frame and are regulated by two upstream promoters, pre-S1 and S [39].

Entry into the hepatocyte involves the low-affinity interaction of S-HBs with heparan sulfate proteoglycan, followed by interaction of the preS1 domain and NTCP. Subsequent internalization of bound virus and NTCP with epidermal growth factor receptor as a cofactor most likely occurs in a clathrin-mediated manner. The viral nucleocapsid is released from the endosome and transported to the nucleus, where viral replication occurs. In the nucleus, S-HDAg recruits and modulates RNA polymerase II to facilitate messenger RNA transcription. After assembly of the viral ribonucleoprotein (RNP) around the HDV RNA genome, the RNP is exported from the nucleus and enveloped by budding into the lumen of the endoplasmic reticulum [39].

The entry of HBV and HDV into hepatocytes constitutes a fundamental step in the establishment, propagation, and persistence of chronic infection. Both viruses exploit a common hepatocellular receptor, the sodium taurocholate cotransporting polypeptide, located on the basolateral membrane of hepatocytes and which physiologically mediates the uptake of bile acid [28,40]. Viral attachment and subsequent internalization are initiated through high-affinity binding of the preS1 domain of the HBV large surface protein to NTCP. This receptor–ligand interaction is likewise co-opted by HDV, reflecting its obligate dependence on HBsAg for virion assembly and hepatocellular entry [8,41,42].

This shared entry pathway represents a highly compelling therapeutic target in the context of HBV/HDV infection. In individuals harboring both viruses, ongoing viral entry facilitates the continual replenishment of infected hepatocytes, thereby sustaining intrahepatic viral reservoirs even in the setting of effective suppression of HBV DNA replication with nucleos(t)ide analogues [43,44,45]. In addition to the hepatotropic role of HDV (with direct cytocidal action involving the small delta antigen), HDV is involved in immune-mediated hepatocytolysis. Thus, persistent viral entry perpetuates antigenic stimulation and immune activation, thereby exacerbating immune-mediated hepatocellular injury, promoting fibrogenesis, and ultimately increasing the risk of progression to cirrhosis and hepatocellular carcinoma [14,17,46].

Bulevirtide, a synthetic lipopeptide derived from the preS1 domain of the HBV large surface protein, acts as a competitive inhibitor of NTCP, thereby blocking the binding of both HBV and HDV to this receptor and effectively preventing de novo hepatocellular infection [28,30].

Unlike agents targeting viral replication or protein synthesis, entry inhibitors act upstream in the viral life cycle, limiting viral dissemination without directly interfering with intracellular viral processes [31,47]. This mechanism is particularly relevant in HDV infection, where viral replication is tightly linked to host cellular machinery and remains largely insensitive to conventional antiviral therapies [9,48]. From a pathophysiological perspective, blocking viral entry has implications that extend beyond virological suppression. By reducing the influx of newly infected hepatocytes, entry inhibition may gradually decrease the overall burden of infected cells, attenuate necroinflammatory activity, and promote stabilization or regression of liver fibrosis [49,50]. These effects are especially relevant in HBV/HDV infection, where disease severity is driven by the combined effects of viral persistence and dysregulated immune responses [10,11].

By selectively blocking de novo infection of hepatocytes, bulevirtide disrupts the propagation of both HBV and HDV at an early stage of the viral life cycle. Importantly, this host-targeting mechanism confers a high genetic barrier to resistance, as it does not exert selective pressure on viral enzymes [31,51].

Moreover, entry inhibition acts synergistically with nucleos(t)ide analogues used for HBV suppression, targeting complementary stages of the viral life cycle without pharmacological overlap [52,53].

Clinical studies have confirmed that bulevirtide-induced blockade of NTCP translates into meaningful virological and biochemical responses, including reductions in HDV RNA levels, normalization of aminotransferases, and improvements in non-invasive fibrosis markers [54,55,56]. Although bulevirtide does not directly eliminate intracellular HBV covalently closed circular DNA (HBV cccDNA) or integrated HBV DNA, its capacity to prevent ongoing viral dissemination represents a fundamental shift in therapeutic strategy, moving from replication control toward modification of disease dynamics [43,57].

## 4. Anatomopathological and Clinical Evidence of Entry Inhibition in HBV/HDV Infection

Clinically, HDV infection can occur in two distinct forms: coinfection and superinfection [7]. Co-infection occurs when HBV and HDV are acquired simultaneously and usually manifests as acute hepatitis, which in several immunocompetent individuals is self-limiting, although it can sometimes progress to fulminant hepatitis with increased mortality [58,59]. In contrast, superinfection occurs when HDV infects a patient already chronically carrying HBV and is associated with a much more severe course, characterized by an increased likelihood of chronicity, rapid progression of liver disease, and a significant risk of cirrhosis and hepatocellular carcinoma [15].

At the cellular and molecular level, the interaction between HBV and HDV is complex and incompletely elucidated. Although HDV depends on HBV for the production of infectious particles, it paradoxically inhibits HBV replication, particularly at the transcriptional level, which frequently leads to reduced levels of HBV DNA in HDV-HBV infection. This inhibition is explained by activation of the host’s immune response and competition for cellular resources. However, this viral interference does not reduce the severity of the disease. On the contrary, HDV exerts direct cytopathic effects on hepatocytes, unlike HBV, which is largely non-cytopathic and causes liver damage predominantly through immune mechanisms [41,60,61].

HDV replication causes the activation of deleterious intracellular mechanisms, including oxidative stress, mitochondrial dysfunction, and endoplasmic reticulum stress, which contribute to apoptosis, necrosis, and liver fibrogenesis [62]. In addition, HDV induces an intense innate immune response with the activation of type I and III interferons and the release of proinflammatory cytokines, such as IL-6 and TNF-α, which causes chronic liver inflammation and accelerates the progression of fibrosis [63,64]. By combining direct cytopathic effects with immune activation and viral interference, HDV profoundly alters the natural course of HBV infection, resulting in a much more aggressive form of liver disease [42,65].

Elevated alanine transaminase (ALT) levels have been associated with long-term complications, such as cirrhosis and hepatocellular carcinoma [21,22,66]. Patients with normal ALT levels had a reduced risk of liver events, including hepatocellular carcinoma (*p* < 0.001) [67].

Clinical studies demonstrated that bulevirtide produces a dose-dependent decline in serum HDV RNA levels, biochemical improvement as evidenced by normalization of ALT values (Table 1) with a favorable tolerability profile, including in patients with advanced fibrosis or compensated cirrhosis [44,52,53]. The observations of 7 clinical studies are summarized in Table 1. The observation of these studies was: a greater than 2 log decrease from baseline in HDV RNA in 54–92% of patients and normalization of ALT in 48.8–74% of patients after 23–144 weeks of treatment.

Adverse effects observed in MYR3 study were headache, dizziness, nausea, pruritus, fatigue, injection site reactions (reaction, erythema, pruritus, inflammation, pain, hematoma, urticaria, abscess, dermatitis, irritation). Grade 3–4 adverse effects were observed, at 96 weeks of treatment, in 18% of patients receiving bulevirtide 2 mg and 16% of patients receiving bulevirtide 10 mg [54]. Asymptomatic, dose-dependent increases in bile acid levels were observed, with a smaller increase in the Bulevirtide 2 mg arm [54].

An Italian, real life study, published in 2025, on 108 patients treated for 6 months with 2 mg/day of bulevirtide noted mild adverse effects, including pruritus (5.6%), injection-site reactions (1.9%) and flu-like syndrome (0.9) [73]. A French multicenter study published in 2025 on a group of 20 patients on the waiting list for liver transplantation (65%, 10% and 25% were Child-Pugh A, B and C, respectively), treated with 2 mg of bulevirtide daily for 48 weeks, of which only 15 completed therapy and twelve patients (60%) underwent liver transplant, noted that no serious adverse events occurred. Three-month transplant-free survival was 76.9% in the bulevirtide group versus 36.7% (*p* = 0.007) in the group without bulevirtide treatment [74].

## 5. Therapeutic Positioning of Entry Inhibition in HBV/HDV Infection

Historically, etiological therapy in HBV-HDV infection began with PegIFN alfa. Current therapy in Europe is either monotherapy with bulevirtide or combination therapy with PegIFN alfa and bulevirtide (Table 2).

The EASL guideline recommends, from 2023, that all patients with chronic hepatitis with HDV, regardless of the degree of fibrosis including compensated cirrhosis, should be considered for antiviral treatment, patients with decompensated cirrhosis should be evaluated for liver transplantation and patients with hepatocellular carcinoma should be evaluated individually for antiviral therapy taking into account therapeutic priorities and prognosis [33].

The AGA 2025 guideline discusses the results of bulevirtide therapy, but the molecule does not have prescription approval in the United States [23].

Bulevirtide should be administered at a dose of 2 mg once daily (every 24 h ± 4 h) by subcutaneous injection, as monotherapy or co-administration with a nucleoside/nucleotide analogue for the treatment of pre-existing HBV infection, associated or not with Peg IFN alfa. The nucleotide/nucleoside analogue is associated in chronic hepatitis with HBV DNA above 2000 IU/mL, in liver cirrhosis at any positive level of viremia and in decompensated cirrhosis irrespective of the presence of detectable HBV DNA [33]. The recommendations of the Italian guideline in 2026 state that treatment with nucleotide/nucleoside analogues should be administered in patients receiving anti-HDV agents, in order to prevent HBV reactivation upon HDV suppression, which expands the EASL 2023 recommendations [33].

The clinical significance of entry inhibition has been further reinforced through combination treatment strategies. Trials investigating higher doses of bulevirtide (10 mg/day) administered alongside Peg IFN alfa have demonstrated superior virological and biochemical outcomes compared with monotherapy, including higher rates of HDV RNA suppression, more frequent normalization of ALT, and more substantial reductions in HBsAg levels (functional cure) [25]. These findings suggest a synergistic interaction between viral entry blockade and immune modulation, with potential implications for functional cure strategies targeting both HBV and HDV. Notably, participants in these studies were consistently maintained on background nucleos(t)ide analogue therapy for HBV, highlighting the complementary role of viral entry inhibition within the context of established HBV treatment strategies.

In light of these observations, in clinical practice, if both PegIFN alfa and bulevirtide are available, combination therapy could be initiated in patients with chronic HBV/HDV coinfection and compensated liver disease. Especially in situations where interferon is not available, or well tolerated, or contraindicated by comorbidities or decompensated liver disease, bulevirtide monotherapy should be initiated. It should also be noted that PegIFN alfa can be used for limited periods of time and bulevirtide can be prescribed for longer periods of time due to its more favorable safety profile. Therapy with nucleotide or nucleoside analogues will begin at the same time as PegIFN alfa-bulevirtide combination therapy or bulevirtide monotherapy [33].

The therapeutic role of bulevirtide is especially pertinent in patients who are suboptimal candidates for interferon-based therapy. Peg IFN alfa, long regarded as the only potentially disease-modifying treatment for HDV, is constrained by limited response rates, mild to moderate tolerance problems, easily managed during limited treatment are more difficult to accept over long periods, and numerous contraindications that restrict its use in routine practice [21,75]. By contrast, bulevirtide provides a host-targeted approach with a favorable tolerability profile and can be administered safely in individuals with compensated cirrhosis, as well as in those who have not responded to or are unable to tolerate interferon therapy [51,76]. This evolution in treatment strategy illustrates a broader shift from immune stimulation toward targeted interruption of key viral life-cycle mechanisms [77].

The duration of treatment with bulevirtide should take into account HDV RNA, ALT, tolerability and the results of future clinical trials. Also, for treatment discontinuation, available data suggest that caution is warranted. The optimal duration of therapy remains uncertain, and continued treatment appears to provide continued benefit, even in patients who do not achieve an early virologic response. In addition, HDV RNA rebound after treatment discontinuation remains a significant concern, particularly in patients with more advanced disease. For this reason, treatment discontinuation should be approached with caution and, in most cases, avoided unless clearly justified by clinical worsening or increasing HDV RNA [33].

Taken together, available clinical evidence supports viral entry inhibition as a disease-modifying strategy in HBV/HDV infection. Although bulevirtide does not eradicate intracellular HBV cccDNA or integrated viral sequences, its capacity to prevent new infection events provides a mechanistically rational complement to HBV polymerase inhibitors and emerging HBsAg-targeting therapies [43,44,78,79].

Looking ahead, inhibition of viral entry offers a rational foundation for combination regimens designed to target multiple, complementary components of the viral life cycle and the host immune response. Potential therapeutic partners include nucleos(t)ide analogues, nucleic acid polymers, small interfering RNA based approaches, and immunomodulatory agents [78,80,81]. Within such multidimensional strategies, bulevirtide serves to restrict ongoing viral dissemination, while additional agents aim to suppress intracellular replication, reduce antigen production, and promote immune reconstitution. This integrated approach parallels combination treatment models that have proven effective in the management of HIV and hepatitis C virus infection [26,82].

## 6. Limitations of Therapy That Inhibits HDV Entry into Hepatocytes

- Long-term durability of response

The durability of response to bulevirtide treatment remains an important and still poorly understood issue in HBV/HDV infection. Data from phase II and III clinical trials, including MYR202 [52], as well as recent studies [54,83] consistently demonstrate that continued therapy is associated with significant reductions in HDV RNA and normalization of ALT levels. However, these benefits are predominantly observed during active treatment. Available studies do not yet provide strong evidence of maintenance of response after discontinuation of therapy, suggesting that the antiviral effect is dependent on continued administration. This observation is consistent with the mechanism of action of bulevirtide. The drug blocks viral entry into hepatocytes without directly affecting intracellular replication or existing viral reservoirs. In addition, the relatively short follow-up duration of most studies limits the assessment of long-term durability of response. Thus, there are currently insufficient data to define the optimal duration of therapy or the likelihood of maintaining response after discontinuation.

The MYR 301 [54] observation was: 90% of adults with chronic hepatitis Delta virus who achieved undetectable HDV RNA at 96 weeks of treatment with bulevirtide remained undetectable for almost 2 years after stopping treatment. The current consensus is still for as long as possible therapy with bulevirtide, due to the clinical observation that once the duration of therapy increases, the percentage of virological and biochemical success increases [54].

- Relapse after treatment discontinuation

There is well-documented data on the occurrence of acute hepatitis after discontinuation of antiviral therapy with Peg-IFN alfa for HBV and HDV [84,85,86]. In these patients, post-treatment exacerbation of hepatitis (defined as ALT elevations of at least 2 times the end-of-treatment values) was observed in 14% of participants in the HIDIT-II study [87].

Exacerbation of hepatitis after discontinuation of bulevirtide treatment was observed in clinical studies MYR202 and MYR203, with 24 weeks of treatment-free follow-up and MYR204, with 48 weeks of treatment-free follow-up. The majority of cases were asymptomatic, did not require liver transplantation, and did not result in death, but are considered serious adverse events. Cases of hepatic decompensation with jaundice, requiring rechallenge with bulevirtide and nucleoside or nucleotide analogues, have also been reported. In study MYR204, a post-treatment ALT value > 5 ULN was observed in 27% of participants who discontinued BLV (2 mg or 10 mg), with or without Peg-IFN alfa, during the 48-week post-treatment period, and 11% of participants had an ALT value > 10 ULN [88].

To prevent this reaction, careful monitoring of liver function, both clinically and laboratory, for at least several months is recommended in patients who discontinue bulevirtide treatment. Reintroduction of bulevirtide and nucleoside/nucleotide analogue therapy may be an appropriate measure to minimize this risk [88].

- Cost and accessibility

The cost and accessibility of bulevirtide are key factors that may limit its implementation in clinical practice, despite its demonstrated efficacy. This is particularly relevant in the context of HDV infection, which is more common in resource-limited settings.

The study published by Buti et al. [89] highlight that therapy, although effective, is costly, especially when it is likely to be administered long-term or even indefinitely. The lack of clear data on the optimal duration of treatment further contributes to the uncertainty regarding the overall economic impact.

The American Gastroenterological Association (AGA) Clinical Practice Update (2025) highlights that the implementation of innovative therapies is influenced not only by efficacy but also by their feasibility in healthcare systems. At the same time, the EASL 2023 guideline shows that although therapy is available in Europe, effective access may vary significantly between countries [23,33].

The epidemiological distribution of HDV accentuates this problem, as the disease is common in regions where resources are limited and access to modern therapies is restricted.

- Limited approval outside Europe

The global availability of bulevirtide remains uneven. Currently, the therapy is approved primarily in Europe and included in the EASL 2023 guideline, which has allowed its integration into clinical practice in Europe [33].

In other regions, access is more limited and depends on local approval processes. The AGA Clinical Practice Update (2025) reflects this situation, highlighting that the use of therapy is conditional on availability in national health systems [23].

The analysis by Kang and Syed [29] shows that the global adoption of bulevirtide is progressive but uneven, generating significant differences between regions in terms of access to treatment. This heterogeneity limits both patient access and the generalizability of clinical data, most of which come from European cohorts.

- Need for combination therapy

The need for combination therapy is an increasingly supported direction in the current literature. Although bulevirtide effectively reduces HDV RNA, clinical trials show that monotherapy rarely leads to HBsAg loss or functional cure [25].

This limitation reflects the fact that blocking viral entry does not directly affect intracellular replication or HBsAg production. In this context, studies reviewed by Gane et al. and Blanchet et al. suggest that therapies that reduce HBsAg or modulate the immune response may complement the effect of bulevirtide [78].

Conceptual analyses support the need for multimodal approaches, targeting multiple steps of the viral life cycle simultaneously [81,82].

Currently, the addition or not of Peg IFN alfa to bulevirtide is used for limited periods of time. Patients with F0-F3 fibrosis and HBV DNA over 2000 IU and those with F4 and positive HBV DNA benefit, according to the Romanian therapy protocol, from the combination of treatment with entecavir or tenofovir. Future research should investigate new therapies that can achieve the clearance of HBs Ag, HBV cccDNA and HDV RNA both at the serum level and in tissue reservoirs [90].

- Lack of data on hard outcomes

Being a new therapy, bulevirtide demonstrates clear virological and biochemical benefits, but the impact over time on major complications such as liver cirrhosis and hepatocellular carcinoma remains to be documented by future studies that will involve long-term monitoring of patients.

Recent observational studies, including those reported by De Ledinghen et al. and Zoulim et al. (2022), confirm short-term efficacy, but emphasize the limited duration of follow-up, insufficient to assess the effects on mortality, HCC or liver transplantation [69,72].

The analysis by Killer et al. (2024) highlights that improvement in surrogate markers cannot yet be directly correlated with reductions in major clinical complications [56].

Patients who had negative HDV RNA levels for two years after discontinuation of treatment were less likely to have: death from liver-related causes (*p* = 0.032) and development of complications (*p* = 0.006) [91].

## 7. Effects on Fibrosis Progression and Clinical Outcomes

Accumulating evidence indicates that sustained HDV suppression under bulevirtide therapy is associated with stabilization or improvement of liver stiffness measurements assessed by transient elastography [55,56,69]. Although histological data remain limited, available biopsy-based analyses suggest reductions in interface hepatitis and lobular necroinflammation following prolonged treatment [49]. These findings support the hypothesis that long-term entry inhibition may modify the natural history of HBV/HDV infection, particularly when initiated before irreversible fibrotic remodeling occurs.

A German study published in 2024, conducted on 15 patients who received Bulevirtide monotherapy for 12 months, observeda decrease in mean liver stiffness from a baseline value of 10.6 kPa to 9.2 kPa at 3 months, 7.8 kPa at 6 months, and then 7.3 kPa at 9 months and 7.6 kPa at 12 months. The difference between baseline and 12 months was statistically significant (*p* ≤ 0.001) [56].

Clinically, slowing fibrosis progression carries substantial prognostic implications. Patients with controlled viral activity are less likely to develop portal hypertension, hepatic decompensation, or hepatocellular carcinoma, complications that historically occur earlier and more frequently in HDV-infected individuals than in HBV monoinfection [14,17,46]. While long-term outcome data are still maturing, early real-world observations suggest a reduction in liver-related events among patients achieving sustained virological and biochemical responses [92].

## 8. Patient-Reported Outcomes and Quality of Life

In addition to improvements in virological and biochemical parameters, bulevirtide therapy has been associated with beneficial effects on patient-reported outcomes. Phase III trials and observational cohorts report improvements in fatigue, physical functioning, and overall health-related quality of life, particularly when compared with interferon-based regimens [51]. These benefits are clinically relevant given the substantial psychological burden, social stigma, and functional impairment associated with chronic HBV/HDV infection [89,93].

The favorable tolerability profile of bulevirtide—characterized primarily by asymptomatic elevations in bile acids rather than systemic toxicity—facilitates long-term therapy and supports sustained patient adherence [83].

In parallel, bulevirtide-based regimens have demonstrated significant improvements in health-related quality of life, reflecting both virological control and the absence of interferon-related toxicity [89,94]. From a health-economic perspective, effective viral suppression may offset high acquisition costs by reducing hospitalizations, delaying decompensation, and lowering transplantation rates, particularly among patients with advanced type of fibrosis [95].

Continued follow-up through prospective longitudinal studies and real-world registry analyses will be critical to clarify the long-term prognostic implications of bulevirtide-based approaches, including their impact on fibrosis regression, prevention of hepatocellular carcinoma, transplant-free survival, and patient-reported quality of life outcomes [57,92].

## 9. Conclusions and Future Perspectives

Bulevirtide, a new class of HDV entry inhibitors, has demonstrated consistent efficacy in improving biochemical and clinical markers of liver inflammation with a favorable safety profile. Observations from the 7 clinical trials analyzed indicate a greater than 2-log decrease in HDV RNA from baseline in 54–92% of patients and a normalization of alanine transaminase in 48.8–74% of patients after 23–144 weeks of treatment, and a significant decrease in liver fibrosis, as measured by Fibroscan, at 12 months of treatment.

The conclusion of our study is that this therapy represents an important leap in the etiological approach to chronic HDV infection and in improving the prognosis of these patients, but future clinical trials are needed to define the criteria for therapy discontinuation and the long-term impact. Future studies are also needed to targeting new therapies that can intervene at other stages of the HDV and HBV life cycle not only to achieve HDV RNA negativity but also HBsAg clearance.

## Figures and Tables

**Table 1:** Summary of clinical trial results.

Study	Design	Number of Patients	Treatment	Duration	Virological Response ^+^	ALT Normalization
MYR 301 [54,68]	A phase 3, multicenter, open-label, randomized	150 adults with chronic hepatitis delta, with or without decompensated cirrhosis	Arm1-Bulevirtide 2 mg Arm2-Bulevirtide 10-mg Arm3-Bulevirtide 10-mg	Arm1-144 weeks Arm2-144 weeks Arm3-96 weeks	Arm1- 74% Arm2- 76% Arm3- 92%	Arm1-59% Arm2-60% Arm3-58%
2021 French multicenter [69]	Prospective and retrospective observational, no randomisatin	77 adults with chronic hepatitis delta, with or without cirrhosis	Bulevirtide 2 mg/day	12 months	68.3%	48.8%
2025 European multicenter [70]	Retrospective real-world	244 adults with cirrhosis (95% Child A)	Bulevirtide 2 mg/day	96 weeks	79%	64%
2023- 16 German centers [55]	Retrospective	114 adults including 59 (52%) with cirrhosis	Bulevirtide 2 mg/day	23 weeks	76%	62%
2025 Multicenter Italian trial [70]	Prospective	108 adults with chronic hepatitis or compensated cirrhosis	Bulevirtide 2 mg/day	6 months	54.6%	significant decrease in ALT and AST
2025 Multicenter Italian study [71]	Prospective	445 adults (74% cirrhosis)	Bulevirtide 2 mg/day	72 weeks	54%	74%
2022 Multicenter French study [72]	Prospective	55 adults(55% cirrhosis)	Bulevirtide 2 mg/day	48 weeks	69%	61%

^+^ Virological response is considered undetected HDV RNA or a 2 log decrease from baseline.

**Table 2:** History of guideline recommendations for etiological therapy in HDV infection.

Guide	Year of Publication	Recommended Medicine
EASL [21]	2017	PegIFN alfa
AASLD [22]	2018	PegIFN alfa
WHO [23]	2021	PegIFN alfa
EACS [27]	2021	PegIFN alfa, Bulevirtide
EMA [32]	2023	PegIFN alfa, Bulevirtide
EASL [33]	2023	PegIFN alfa, Bulevirtide
AGA [23]	2025	PegIFN alfa, Bulevirtide

## Data Availability

The original contributions presented in this study are included in the article. Further inquiries can be directed to the corresponding authors.
